# Simulation study of a coincidence detection system for non-invasive determination of arterial blood time-activity curve measurements

**DOI:** 10.1186/s40658-020-00297-9

**Published:** 2020-05-07

**Authors:** Yassine Toufique, Othmane Bouhali, Pauline Negre, Jim O’ Doherty

**Affiliations:** 1 Advanced Scientific Computing Center, Texas A&M University at Qatar, Doha, Qatar; 2 Qatar Computing Research Institute, Hamad Bin Khalifa University, Doha, Qatar; 3 Clinical Imaging Research Centre, Centre for Translational Medicine, National University of Singapore, Singapore, Singapore

**Keywords:** Arterial sampling, Arterial input function, PET, Kinetic modeling

## Abstract

**Background:**

Arterial sampling in PET studies for the purposes of kinetic modeling remains an invasive, time-intensive, and expensive procedure. Alternatives to derive the blood time-activity curve (BTAC) non-invasively are either reliant on large vessels in the field of view or are laborious to implement and analyze as well as being prone to many processing errors. An alternative method is proposed in this work by the simulation of a non-invasive coincidence detection unit.

**Results:**

We utilized GATE simulations of a human forearm phantom with a blood flow model, as well as a model for dynamic radioactive bolus activity concentration based on clinical measurements. A fixed configuration of 14 and, also separately, 8 detectors were employed around the phantom, and simulations were performed to investigate signal detection parameters. Bismuth germanate (BGO) crystals proved to show the highest count rate capability and sensitivity to a simulated BTAC with a maximum coincidence rate of 575 cps. Repeatable location of the blood vessels in the forearm allowed a half-ring design with only 8 detectors. Using this configuration, maximum coincident rates of 250 cps and 42 cps were achieved with simulation of activity concentration determined from ^15^O and ^18^F arterial blood sampling. NECR simulated in a water phantom at 3 different vertical positions inside the 8-detector system (*Y* = − 1 cm, *Y* = − 2 cm, and *Y* = −3 cm) was 8360 cps, 13,041 cps, and 20,476 cps at an activity of 3.5 MBq. Addition of extra axial detection rings to the half-ring configuration provided increases in system sensitivity by a factor of approximately 10.

**Conclusions:**

Initial simulations demonstrated that the configuration of a single half-ring 8 detector of monolithic BGO crystals could describe the simulated BTAC in a clinically relevant forearm phantom with good signal properties, and an increased number of axial detection rings can provide increased sensitivity of the system. The system would find use in the derivation of the BTAC for use in the application of kinetic models without physical arterial sampling or reliance on image-based techniques.

## Background

Arterial blood sampling has long been performed for quantitative PET (positron emission tomography) studies in order to measure the arterial input function (AIF). The process involves the determination of time-activity curves of the PET radiotracer in whole blood (blood time-activity curve—BTAC) and further processing to determine the plasma time-activity curve (PTAC) depending on the subject hematocrit and potential presence of radiolabeled metabolites [1]. The current gold standard technique for determination of the BTAC requires the extraction of arterial blood for on-line real-time radioactive counting via an indwelling cannula in the radial artery which can be an expensive and laborious process requiring the use of skilled clinicians owing to the invasiveness of the procedure. The procedure presents potential clinical complications such as infection, pseudoaneurysm, sepsis, and ischemic damage [2], although safety aspects of over 1000 radial artery cannulations in a population of subjects specifically undergoing PET scanning found a relatively low rate of complications of 0.09% [3]. Although the process is seen as safe, arterial cannulation often discourages patients and subjects from taking part in PET studies and can lead to study cancelations due to technical considerations with arterial cannulation. Furthermore, skilled clinicians are required to place the cannula, adding to study costs.

Non-invasive approaches of determining the BTAC have also been developed, with the most common being the use of an image-derived input function (IDIF) which can be determined through dynamic framing and reconstruction of the images of the initial phases of the radiotracer injection [4]. For certain studies, this IDIF can be obtained by the use of regions/volumes of interest in large vessels or blood pools where partial volume effects are minimal such as the aorta or the left ventricle. However, in studies where large vessels and blood pools are not in the field of view (i.e., in brain studies), regions of interest in smaller vessels (on the order of the scanner resolution) must be used, requiring more complex processing methods for correcting for the effects of partial volume and the associated underestimation of peak activity. Blood samples may also still be required if there is metabolism of the tracer during the scan. There are many works detailing methods for segmenting vessels in the head and neck and employing various partial volume processing techniques such as the use of recovery coefficients [5], MR-guided segmentation [6], factor analysis [7], and dispersion modeling [8]. Recent work has concluded that although IDIF can be successfully implemented with only a minority of PET tracers, it is not a “one-size-fits-all” approach to kinetic modeling and remains logistically challenging [9, 10].

Population-based input functions (PBIF) have also gained interest in order to remove the reliance on invasive arterial measurements. The technique is based on the individual scaling of an already defined tracer-specific input function of standard shape, and scaling can be performed a number of ways. For example, if blood is extracted for scaling, the technique typically requires a much reduced number of venous blood samples over the scan length [9–11]. Although not affected by issues with image quality or resolution, PBIF is usually represented by a mathematical function, and its biggest limitation is that the input function shape determined in a group (i.e., in healthy subjects) may be different to that of another group (i.e., disease-affected patients), because of altered metabolism/uptake of the radioligand, and hence does not take individual variation into account. Furthermore, the accuracy of PBIF depends on the metabolite fraction of the tracer, and previous work has shown issues in the accuracy of this method, such as for ^11^C-PBR28 due to low parent fraction of the tracer at the end of the scan [7].

Tissue uptake functions have also been employed which have the potential to avoid any measurement of the BTAC from images or from the blood. A recent study quantifying cerebral blood flow in 29 subjects using ^15^O-H_2_O employed a technique whereby the BTAC can be simulated using the tissue uptake function (from imaging) and a rate constant [12, 13]. The estimated difference between measured and simulated BTAC and associated parametric maps were approximately < 10%. Although the technique seems promising, it requires the calculation of a minimum of 500 tissue uptake curves to enable the simulation and thus requires significant resources and experience to operate.

Previous hardware solutions have also investigated non-invasive methods of determining the BTAC. Initial work some years ago investigated a dual plastic scintillation system arranged to detect gamma emissions from the wrist [14], while more recent work investigated a prototype system consisting of 2 detector pairs of LSO and avalanche photodiodes (APDs) to obtain images of wrist phantoms with good spatial resolution and sensitivity [15]. Further in vivo investigations and simulations showed the ability to discriminate between arterial and venous flow [16], and the group also developed a 10-cm-diameter closed ring 3D tomographic system which correlated well to gold standard blood sampling measurements [17]. Determination of the BTAC in their work required the generation of high-resolution images, acquired using a grid of 4 × 8 LYSO crystals and associated avalanche photodiode (APD) module, and the use of ROIs to detail the whole blood input function. Recent work with phantoms has also investigated the use of prototype system using polystyrene-based scintillating fibers and silicon photomultipliers (SiPM) with a venous access catheter. The system has shown good promise in terms of linearity, sensitivity, and signal-to-noise [18]. Similar work developed a PET-CT system designed to image rheumatoid and psoriatic arthritis in the wrist and the hands of patients, although as yet this system focuses only on high-resolution imaging of the joints of the wrist rather than detailing BTAC [19].

This work similarly investigates the feasibility of circumventing issues with both invasive catheterization of the radial artery and also complex processing techniques associated with IDIF calculation in imaging fields that suffer from partial volume effects by the use of an external detection system aimed at non-invasively measuring the BTAC in vivo.

## Methods

### GATE Monte Carlo simulation software

Several Monte Carlo codes have been developed to simulate the interaction between radiation and matter. There is a wide range of developed codes such as BeaMnrc [20], FLUKA [21], MCNP [22], PENELOPE [23], and GEANT4 [24]. GATE (Geant4 Applied for Tomographic Emission) is a Monte Carlo code based on GEANT4 [25]. It includes specific modules required to perform realistic simulations of imaging technology and offers a complete set of validated physical models, description of complex geometries, description of the source motion and geometry, generation and monitoring of particles, and visualization of volumes and particle trajectories. However, as is the case with many Monte Carlo platforms, simulations require significant computing resources. In this work, we employed the “Raad2” supercomputer located at Texas A&M University Qatar, to run all GATE simulations. The system is comprised of a Cray XC40-AC supercomputer (Cray Inc., Seattle, USA) with 4128 Intel Xeon Haswell cores, containing 172 computation nodes, each one containing 24 cores, along with 128 GB of RAM. It is served by a Lustre shared storage file system (available under GNU general public license v2) with 800 TB capacity and uses the SLURM (Simple Linux Utility for Resource Management) software (available under GNU general public license v2) to allocate and manage computational resources.

### Phantom design

**Fig. 1:**
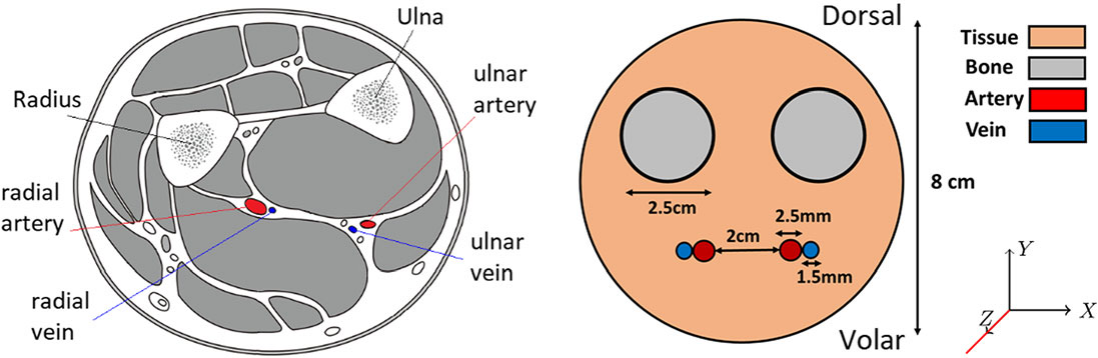
Left—cross section of the lower forearm (left) detailing the anatomical location of the vessels and bones near the wrist. Right—schematic diagram showing a cross section of the basic wrist phantom (not to scale)

A basic phantom was designed based on MR (magnetic resonance) images of the human forearm. The phantom consisted of a cylinder 20-cm long and 8 cm in diameter. Two cylinders of 2.5 cm diameter, representing the radius and ulna bones (given the physical properties of bone in GATE simulations), were designed parallel to the central axis and separated by a distance of 1.1 cm. Two other cylinders of 2.5 mm diameter representing respectively the radial and ulnar arteries (given the physical properties of blood) were simulated (Fig. 1). Cylinders of 1.5-mm diameter representing the radial and ulnar veins were also included. The background tissue in the phantom was given the properties of water. The phantom was centered in the scanner geometry on simulation.

### Scanner configuration

**Fig. 2:**
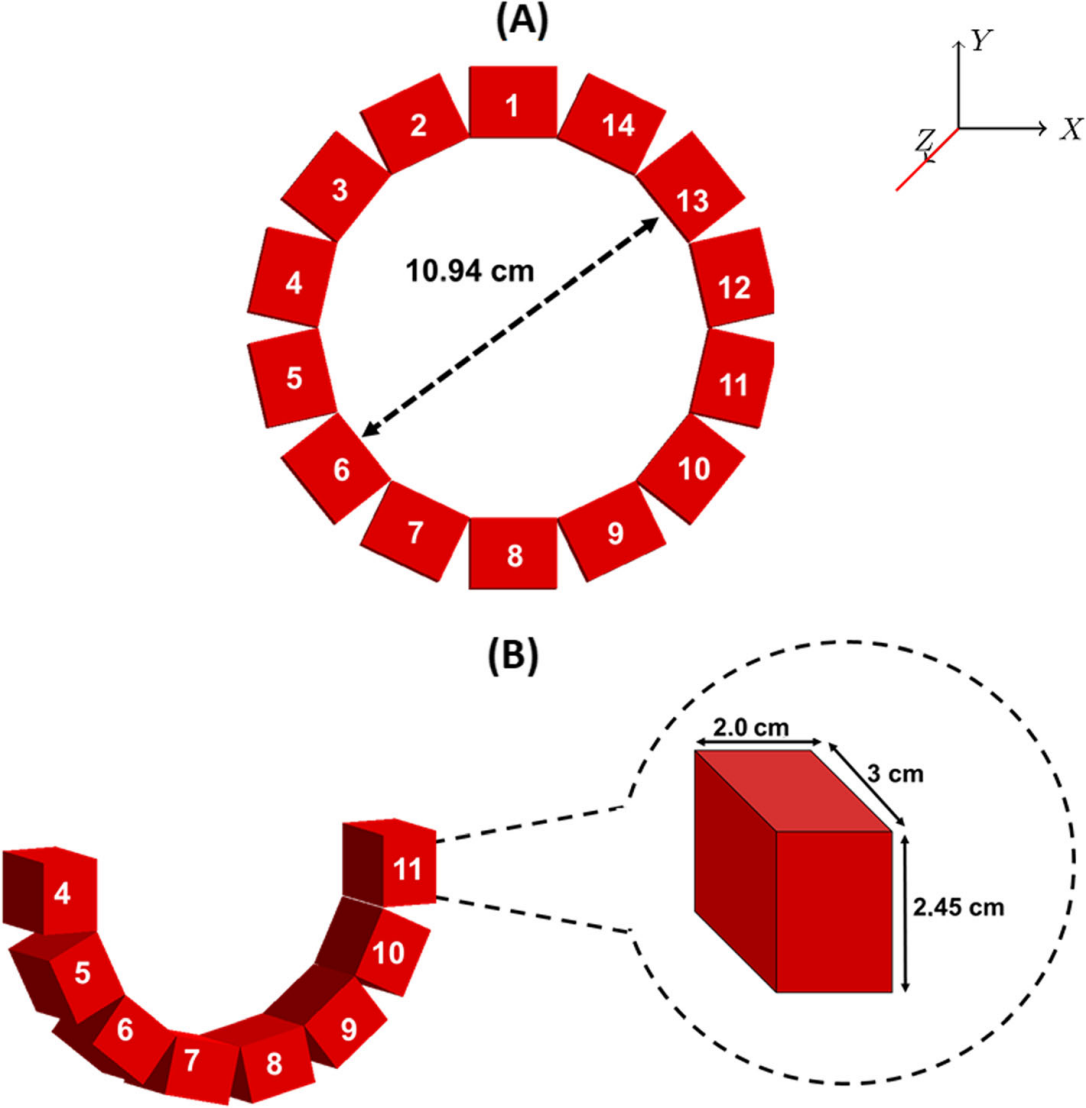
Schematic detailing layout of the wristPET1 (**a**) system. wristPET1 consists of 14 detectors in a closed ring design, whereas a second system (wristPET2 (**b**)) was projected to employ an open-ring design with a subset of detectors (numbers 4**-**11) only. All other properties of the detectors between wristPET1 and wristPET2 are identical

The first simulated scanner (called “wristPET 1”) consisted of 14 detectors organized in a single ring with a 3-cm field of view. Each detector consists of a rectangular monolithic scintillation crystal (Fig. 2) measuring 2.45 × 3.0 × 2.0 cm^3^. The ring diameter was chosen to fit the dimension of human wrist circumference based on a survey of 20 MRI wrist scans at our center. Five crystal materials were simulated given their use in clinical and research PET detection technology in literature (Table 1): BGO (bismuth germinate), GSO (gadolinium orthosilicate), LSO (lutetium orthosilicate), CeBr_3_ (cerium bromide), and LaBr_3_ (lanthanum bromide), and simulations were performed to verify the material with the highest efficiency for low activity detection. In all simulation cases, the digitizer module converting hits to singles and coincidences which consisted of a series of signal processors (Adder, readout, blurring, deadtime, energy response, spatial response, threshold electronics, and deadtime) were employed. Default parameters of the GATE platform were utilized for the selected crystal material for variables such as energy resolution and energy window (Table 1). Coincidence time employed was manually set to the literature times outlined in Table 1. Scatter and random corrections were applied to the resulting coincidence count rate data using the “KeepIfAllAreGood” policy of the coincidence sorter. The isocenter of the detection system was assigned the coordinated (*X* = 0 cm, *Y* = 0 cm, *Z* = 0 cm).

**Table 1:** Selected properties of the scintillator materials used in this work

Property	GSO (Ce)	LSO (Ce)	BGO	CeBr3	LaBr3(Ce)
Density (g/cm^3^)	6.71	7.40	7.13	5.20	5.30
*Z*_eff_ @ 511 keV	58.6	65.0	74.2	45.9	46.9
Decay time (ns)	60	40	300	17	26
Wavelength (nm)	430	420	480	370	370
Light output (Photons/keV)	12-15	20-30	8	60	70
Index of refraction @ wavelength	1.85	1.82	2.15	1.88	1.88
Energy resolution @ 511 keV	8.5	10.0	10.2	5.2	3.2
Attenuation length @ 511 keV (mm)	14.3	11.4	10.4	19.6	24.0
Coincidence windows time (ns)	4.5	4.5	10.0	4.5	4.5

*GSO* gadolinium orthosilicate, *LSO* lutetium oxyorthosilicate, *BGO* bismuth germanate, *CeBr3* cerium bromide, LaBr3 lanthanum bromide. Table after [26, 27]. *Z*_eff_ represents the effective atomic number and stopping power can be calculated as the inverse of the attenuation length

### Modification of scanner

**Fig. 3.**
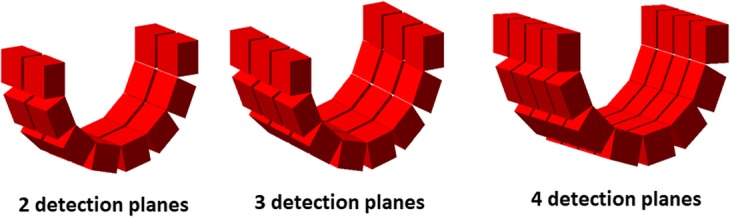
Schematic of the wristPET2 model with 2 (6 cm FoV), 3 (9 cm FoV), and 4 (12 cm FoV) total axial rings f detectors

Due to the anatomy of the larger blood vessels on the volar aspect of the wrist (Fig. 1) that create the majority of the BTAC signal (radial and ulnar arteries), we also hypothesized that an open-ring system consisting of a subset of detectors only, detectors 4–11, and designated “wristPET2” (Fig. 2b) would also produce count rates acceptable to the determination of the BTAC. We performed simulations of the count rate capabilities for the wristPET2 system using a decaying point source experiment, with a 1-ml point source of activity (14 MBq of ^18^F at starting time) located within an 8-cm-diameter water-only phantom, entirely in the FoV of the system in 3 different positions corresponding to where the arteries may be found. The origin of the detection system remained as the same point as for the wristPET 1 system. The point source (1 mm^3^) was simulated at the position of (*X*, *Y*, *Z*) = (0 cm, − 1 cm, 0 cm), (0 cm, − 2 cm, 0 cm), and (0 cm, − 3 cm, 0 cm) and the count rates determined as the source decayed. NECR (noise-equivalent count rate) was calculated with the standard equation:

$$ \mathrm{NECR}=\frac{T^2}{T+S+\left(k\times R\right)} $$

where *T*, *S*, and *R* represent the number of true, scatter, and random coincidences respectively and *k* = 1 for this configuration. Furthermore, we also performed simulations of added detection rings in the axial direction (2, 3, and 4 rings) of wristPET2 in order to investigate the increased sensitivity of the system (Fig. 3).

We also investigated differences in sensitivity due to the use of different radiotracers for ^18^F and ^15^O; given that these radiotracers are commonly used in brain studies where large vessels or blood pools are unlikely to be in the imaging field of view and cannot be used for image-based input functions. The use of these radiotracers for imaging studies nominally uses different levels of injected activity of approximately 250 MBq and 1000 MBq for ^18^F and ^15^O respectively.

### Blood flow models

In order to add clinical relevance to the simulations for the detection of the BTAC, the addition of dynamic changing of activity concentration was implemented. Two models of blood flow through the arteries were investigated. Firstly, for the wristPET1 system, a uniform blood flow through the arterial vessels of the phantom was simulated (using a mean value of 15 cm/s determined from literature [28]) as a basic test in order to observe crystal response. There was no venous return blood for this simulation, and this was the only simulation performed with wristPET1.

Secondly, for wristPET2, a more realistic pulsatile blood flow model was investigated, whereby the cylinders representing the arteries with uniform activity concentration were moved through the scanner in a pulsatile motion according to a measured velocity profile of blood through the radial artery from literature [29]. We assumed a standard heart rate of 60 bpm for repetition of the pulses. For all simulations using the wristPET2 system, arterial blood passed through both arteries as defined above, and in order to simulate venous return of blood from the capillary bed of the hand, the blood began to return in the opposite direction through the radial and ulnar veins of the phantom at a uniform velocity of 5 cm/s at a reference time of 5 s later [30].

In both blood flow models, changes in the activity concentration for providing a simulation of the BTAC were achieved by employing a model linear-exponential equation. The equations were fitted to BTACs acquired from patient studies on a fully calibrated automated blood sampling and counting system (Allogg AB, Sweden) acquiring extracted arterial blood activity concentration after an injection of 1000 MBq of [^15^O]-H_2_O and 250 MBq [^18^F]-fallypride at 1 s intervals. Two average BTACs were created from 5 samples of these tracers respectively, and the series of equations used to fit to these two averages can be summarized as [31]:

$$ \mathrm{AIF}(t)=\left\{\begin{array}{c}0\  for\ t\le {t}_0\\ {} at+b\  for\ {t}_0<t<{t}_1\\ {}{c}_1\bullet {e}^{-{d}_1(t)}+{c}_2\bullet {e}^{-{d}_2(t)}\  for\ t\ge {t}_1\end{array}\ (1)\right. $$

Parameters used for the fit of the true [^15^O] H_2_O BTAC (*r*^2^ = 0.99) and for the [^18^F]-fallypride fit (*r*^2^ = 0.98) are detailed in Table 2. These fitted noise-free BTACs were then used as input to the radial and ulnar arteries of the phantom, for the specific radiotracer with the activity concentration of the arteries of the phantom varied according to the activity concentration as determined from Eq. 1. The function describing volumetric flow per unit time was implemented along with the function describing the changing activity concentration of the sources as described by Eq. 1.

**Table 2:** Fit parameters for arterial and venous data fits to [^15^O]-H_2_O and [^18^F]-fallypride data using Eq. 1

	Arterial data fit		Venous data fit	
Parameter	[^**15**^O]-H_2_O	[^18^F]-fallypride	[^**15**^O]-H_2_O	[^18^F]-fallypride
a	9.248	2.956	2.606	1.291 × 10^−1^
b	1.535 × 10^3^	− 4.907 × 10^2^	− 4.458 × 102	− 2.209 × 10^1^
c1	1.959 × 10^5^	6.088 × 10^4^	1.810 × 105	1.197 × 10^4^
c2	2.061	2.059	2.060	2.060
d1	− 3.902 × 10^−2^	− 3.902 × 10^−2^	− 3.902 × 10^−2^	− 3.902 × 10^−2^
d2	− 8.271 × 10^−5^	− 8.271 × 10^−5^	− 8.271 × 10^−4^	− 8.271 × 10^−4^

Equation 1 was also employed to generate dispersed (broadened peak with concomitant loss of peak height) and delayed (time shifted) venous output functions with the following parameters. Parameters for the fits to these functions for [^15^O]-H_2_O and [^18^F]-fallypride data are detailed in Table 2. Fits for both curves were both *r*^2^ > 0.98.

## Results

### Crystal type selection

**Fig. 4:**
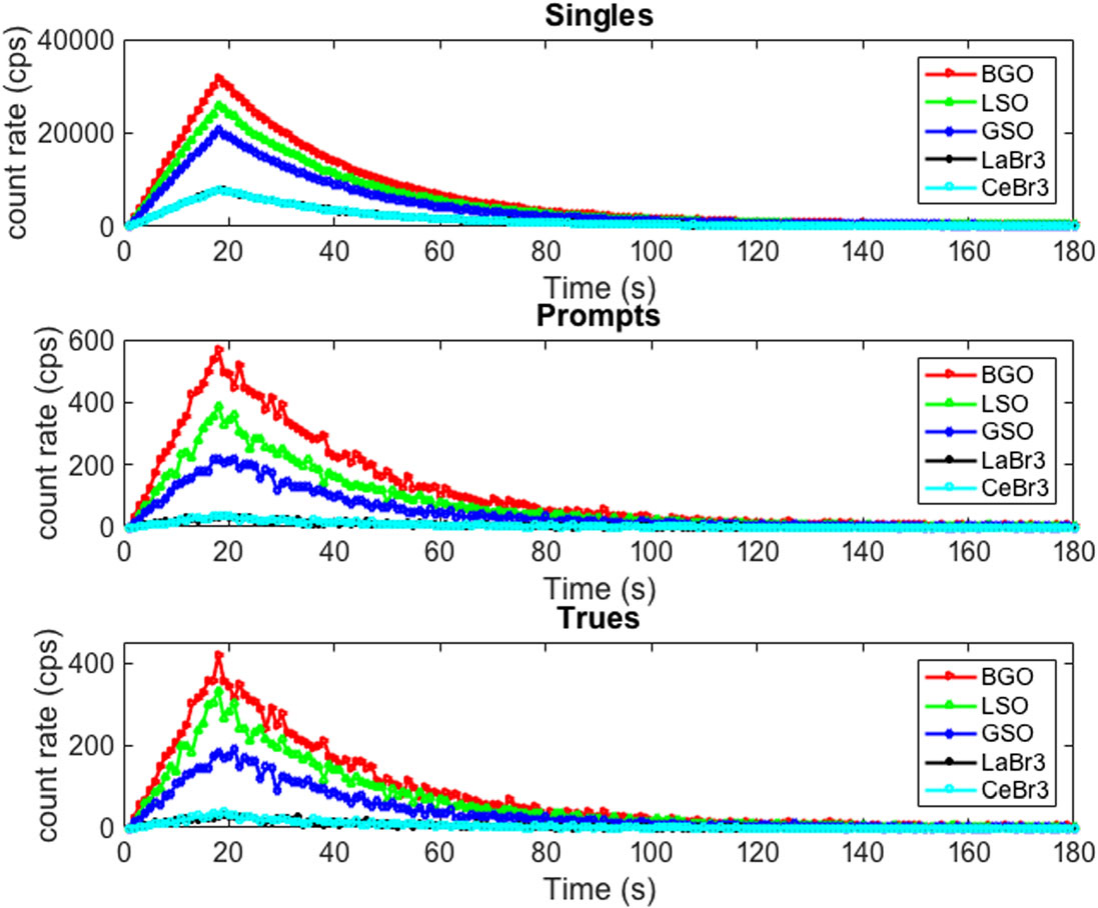
Single, prompt, and true count rates for each crystal material used in the simulation of a physically recorded BTAC using wristPET1. All simulation criteria were constant except the crystal material and associated parameters from Table 1. The highest count rates for both singles and coincident rates can be noted for BGO crystals. There was no venous return of blood in this simulation, only arterial blood at a uniform velocity. Similar responses can be noted for LaBr3 and CeBr3 crystals and they appear as overlapping on the figure

Singles and coincidence rates were obtained from the wristPET1 system when using each of the five crystal materials considering single sources of radiation traveling through the ulnar and radial arteries only (Fig. 4). It can be observed that the highest counts for singles and coincident events are for BGO crystals. This agrees well with previous experimental work using low activity sources on clinical scanners demonstrating the improved performance of BGO crystals over LSO [32]. Therefore, BGO was employed as the crystal material for all subsequent simulations using the wristPET2 system.

### Rationale for generation of wristPET2

As described above, the anatomical position of the blood vessels transporting blood through the forearm/wrist of humans is primarily towards the volar aspect of the forearm. Thus, in the simulated phantom (and the position of the phantom inside the scanner), plotting the total number of singles on the detectors of wristPET1 over an entire simulation demonstrates that a high number of singles are observed in detectors 4-12. For a single simulation, there were 1.42 million counts for wristPET1 versus 1.03 million for wristPET2 under identical conditions, and on average, between 70-75% of counts are observed in these detectors. A similar effect is noted in PET myocardial perfusion scans during the initial phases of dynamic imaging owing to the proximity of the heart to the top of a full ring clinical PET scanner [33, 34]. Thus, given that approximately 25-30% of singles are incident on the remaining detectors of wristPET1, our hypothesis that they can be removed from the design without significant reduction in the count statistics shows that wristPET2 was a good alternative to a full-closed ring system. As BGO showed the highest sensitivity and for other reasons involving low cost of the material for potential construction of the system described in the “Discussion” section, this material was implemented in wristPET2 and all other materials excluded.

### Count rate performance

Simulations were performed investigating the count rate performance of wristPET2 using the decaying point source experiment detailed above. Due to the configuration of the detector, the count rates increase as the source moves more ventral in the scanner due to greater coverage by the detectors. NECR_max_ determined for each vertical location is 8360 cps (*Y* = − 1 cm), 13041 cps (*Y* = − 2 cm), and 20,476 cps (*Y* = − 3 cm) at an activity of 3.5 MBq. At *Y* = − 3 cm, the trues ratio is increased by a factor of 2.3 and the maximum (NECR_max_) is increased by a factor of 2.5 compared to the *Y* = − 1 cm situation.

### BTAC simulations with wristPET2 system

Simulations were performed with the wristPET2 system using fits to measured BTAC corresponding to injections of approximately 200 and 1000 MBq of ^18^F and ^15^O respectively in order to evaluate the characteristics of the wristPET2 system to high and low count rates.

Simulations were performed to compare the count rates between in the case of ^18^F and ^15^O simulations, inclusive of the arterial pulsation and BTAC mathematical models (Fig. 6). An overlap of the signals from the venous and arterial functions can be observed, in these cases with only a small contribution from the veins. In both cases (^18^F and ^15^O), the coincident rates show that the BTAC can be described by the system. Less noise is noted for the BTAC described due to ^15^O as the activity used in the simulation was 4 times higher than that of ^18^F.

### Number of detection rings

Simulations were performed to investigate the sensitivity of wristPET2 by adding 1, 2, and 3 extra rings of detectors, allowing fully 3D coincidences between the rings. Count rates were largely enhanced by the addition of extra detection rings, leading to higher overall sensitivity of the system (Fig. 7). Gains of over a factor of 5 were obtained for an increase of 1 to 4 rings of similar monolithic crystals, and the summation of the arterial and venous return curves can be clearly observed in the simulations of both ^15^O and ^18^F. Furthermore, the sensitivity can be seen to increase in a linear manner with the addition of extra detection rings (Table 3).

**Table 3:** Gain in singles rates, coincident rates, and sensitivity for the wristPET2 system with increasing number of detection rings. The reference values in these cases are the values determined from the single half-ring 8-detector system

Amount of rings	Max singles rate gain (@ peak)	Max coincidences gain (@peak)	Sensitivity (singles) (cps/kBq)	Sensitivity (coincidents) (cps/kBq)
^15^O				
2	2.64	8.84	230.11	4.12
3	4.87	33.27	424.1	15.52
4	7.29	72.73	635.1	33.94
^18^F				
2	2.63	12.10	231.85	2.87
3	4.90	49.10	431	11.68
4	7.40	109.0	650.43	25.93

## Discussion

Our work has described the theoretical simulation of a coincidence detection system which can be used for in vivo determination of the blood time-activity curve (BTAC) in PET studies. The simulated detection system was tested with point sources for determination of count rate performance and also with a dedicated wrist phantom (Fig. 1), which provided the capability for a radioactive bolus of blood to move through two arteries and return through two veins, with a model included for the pulsation of the blood. We proposed a single ring detection setup and a further modification of a single ring design to a subgroup of 8 detectors owing to the geometry of the human wrist and the location of the blood vessels providing the signal (Fig. 2). Further optimization of the system included the investigation of the number of detection rings to provide an increase in sensitivity (Fig. 3).

There are a number of aspects to be considered for the development of the hardware of such a system such as the wristPET2. For example, the choice of a scintillating material for the application described in our work is a tradeoff between different important parameters such as decay time, material density, and thickness. For the requirements of producing a BTAC, we propose that generation of an image may not be required, and therefore, compromises on the choice of scintillation crystal can be made. The count rates from a simulation using a generated synthetic BTAC with a range of scintillating materials and details BGO as the best performing scintillator in terms of singles and coincident count rates (Fig. 4). As outlined in Table 1, the materials CeBr_3_ and LaBr_3_ have similar properties in terms of light output, density, and decay time, and hence, the curves of singles and coincident rates overlap, and the crystals show similar sensitivities in Table 3 and efficiency compared to BGO.

It is known that LSO has better properties as a scintillator for imaging purposes at high activity, mainly related to the much shorter decay time and higher light output [35]. This allows novel electronic collimation techniques such as time-of-flight imaging, in turn leading to higher signal-to-noise and noise-equivalent count gain, shorter coincidence time window (in turn reducing the randoms rate), and a general increase of the count rate capabilities of the detector system. However, in the case of determination of BTAC, we propose that as the generation of an image may not be required, crystals with higher decay time and longer coincidence time window (at the expense of higher randoms rate and longer decay time such as BGO) can provide adequate performance for signal detection purposes. A higher decay time of the scintillator is acceptable due to the expected activity concentration (and thus the count rate) being lower than that measured for example in the left ventricle due to dilution of the radiotracer by the time the bolus reaches the position of the radial and ulnar arteries. In this case, the tradeoff for higher detector efficiency is warranted, with a relatively low risk of encountering issues with deadtime losses. Monolithic BGO crystals are also desirable because of lower cost implications for fabrication of the system, a higher photoelectric fraction (percentage of photons interacting by the photoelectric effect), and lack of intrinsic radioactivity (present in some LSO crystals depending on the crystal growing technique). Our crystal selection choice corresponds well to other work who adopted BGO as the crystal of choice for small animal imaging where low activity concentrations are expected [36, 37].

The rationale for the generation of wristPET2 (Fig. 2), identical to wristPET1 except for the removal of 6 detector modules located at the top of the system, was deemed feasible due to the location of the radial and ulnar arteries in the phantom with an acceptable loss of singles (Fig. 4). We proposed that a fully developed system for human use would also function similarly owing to the fairly repeatable anatomical location of the radial and ulnar arteries. Thus, the cost of a single ring system can be reduced even further by the removal of 6 detectors.

**Fig. 5:**
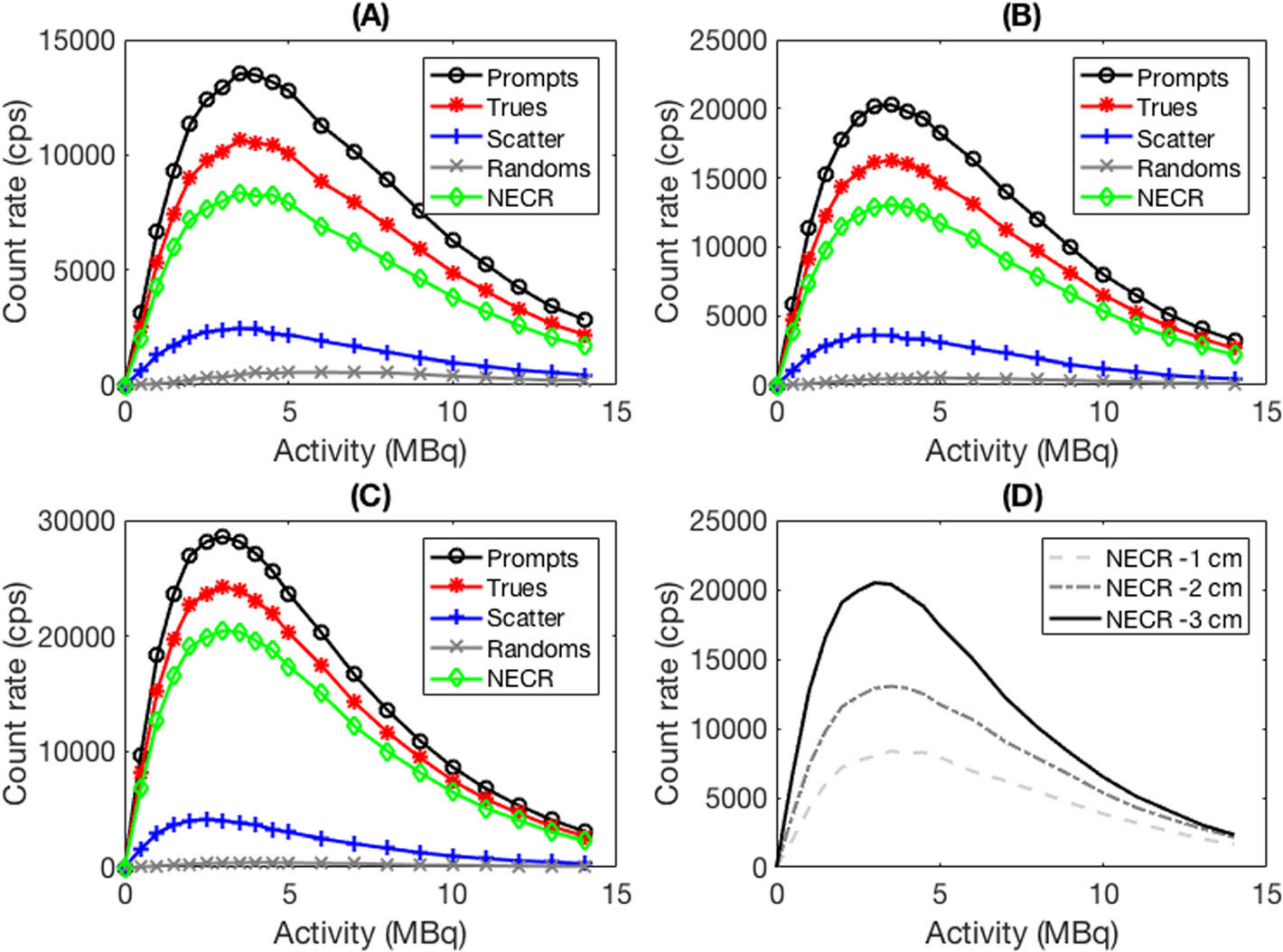
Count rate performance of the wristPET2 (8 detector system) using a point source at different *Y*-positions inside an 8-cm-diameter water phantom at **a***Y* = − 1 cm below center, **b***Y* = − 2 cm below the center, and **c***Y* = − 3 cm below the center of the scanner **d** comparison of NECR at *Y* = − 1 cm, *Y* = − 2 cm, and *Y* = − 3 cm where NECRmax is 8360 cps, 13041 cps, and 20,476 cps at an activity of 3.5 MBq

A low number of scatter and random events were noted in simulating point sources within the phantom as noted in the NEC curves (Fig. 5). Similar low numbers of scatter and randoms have previously been noted on other monolithic crystal detection systems, which also investigated a reduction in the number of blocks to their coincidence detection system [38]. Typical injected activities for PET studies are in the range of 200-1000 MBq; however, by the activity concentration reaching, the radial and ulnar arteries are more likely in the range of < 500 kBq/ml. Thus, at this activity, the system operates within the linear region of the count rate curves (Fig. 5). For our system, the NECR is a function of the vertical position inside the scanner and is also a factor in many anatomy-dedicated systems such as breastPET imaging, and we expect that for routine use, the blood vessels would lie in the region of *Y* = − 2 cm to − 3 cm below the center of the scanner.

**Fig. 6:**
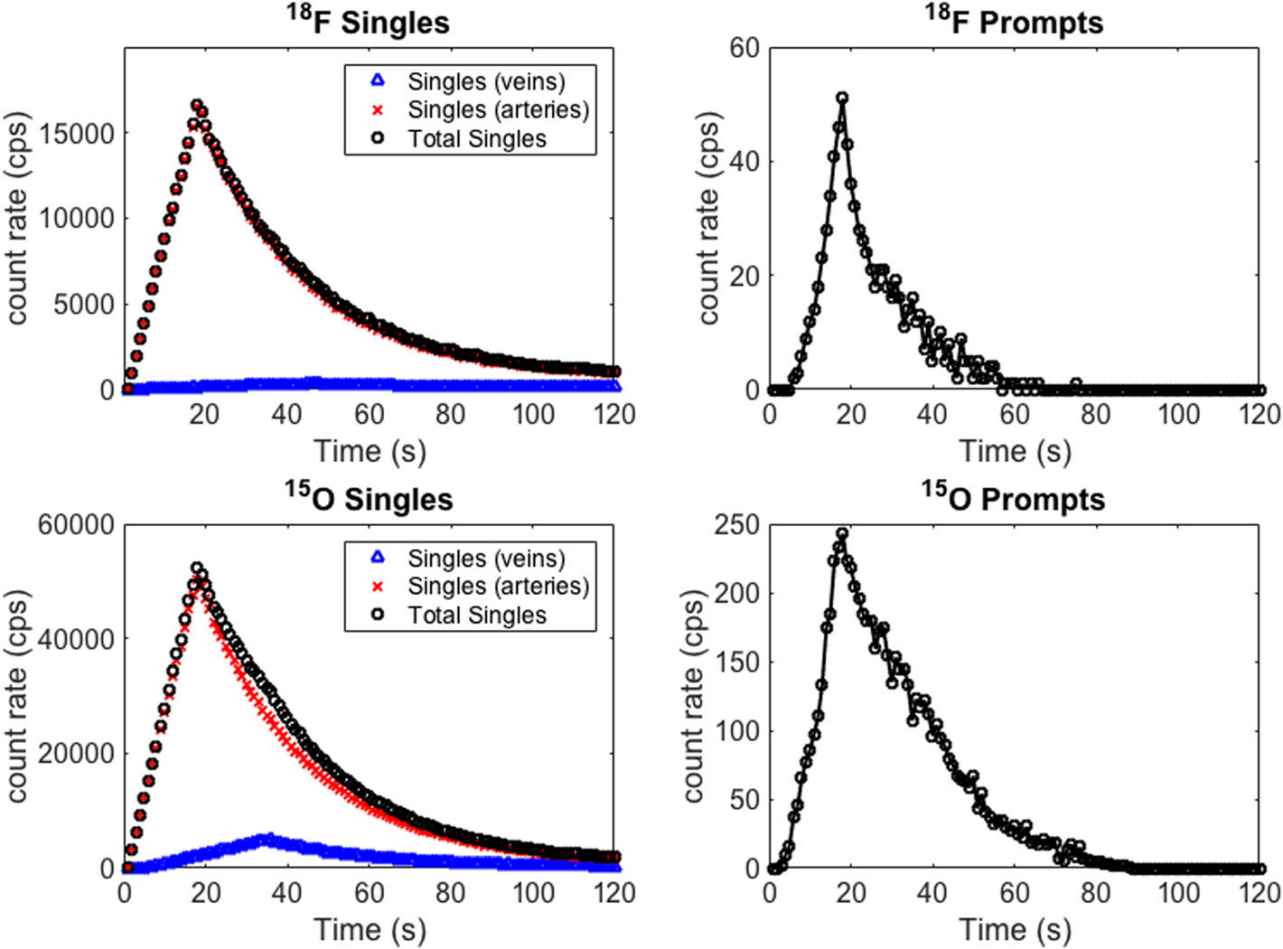
Singles and prompt count rate of a pulsatile flow model using models of BTAC for ^18^F (top row) and ^15^O (bottom row) activities through the simulated wrist phantom. Noise in the prompt rates can be observed with the decreasing counts

Employing this system of 8 crystals, the inclusion of the pulsatile motion model, and the inclusion of a venous output function leads to simulations that represent a more clinical scenario. The overlapping of the arterial and venous phases of the total signal (Fig. 6) can be explained by the delay built into the simulation of the bolus traveling through the arteries and returning through the veins of the phantom. Thus, the total singles and coincident rates include combinations from both arteries and veins; however, the majority arise from the arteries. The coincident rates for the ^18^F simulation are noisier than the ^15^O simulation due to the count statistics from a lower simulated activity but can still be fitted with a function to use as input to a kinetic model.

**Fig. 7:**
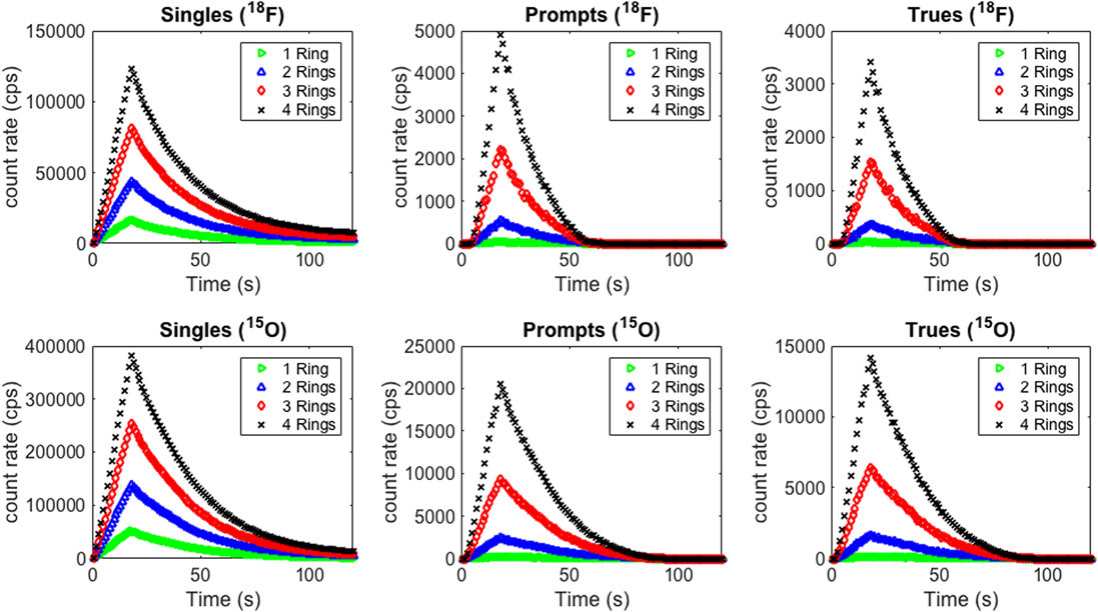
Total singles, prompt, and true event rates for wristPET2 system simulated for an increasing number of detection rings (1, 2, 3, and 4) for ^18^F simulation (top row) and ^15^O simulation (bottom row). All variables were constant except the number of detection rings

An increase in the number of axial detection rings (Fig. 3) leads to a large gain in the number of singles and thus coincidences. As detailed in Table 3, increases in sensitivity result from the addition of further rings (compared to the single ring half-ring system) for simulations of both radiotracers through the phantom. Thus, there is a tradeoff to be achieved between the cost of the proposed system and the desired sensitivity, although our simulations of a full single ring system show sufficient signal from the simulation to allow detection of the BTAC (Fig. 7). It should be noted also that with 4 axial detection rings, this system gives both single and coincident counts comparable to current commercially available on-line blood extraction systems.

### Study limitations and future improvements

Given that the results presented in this work are simulations of the generation of BTAC in human subjects, they therefore do not capture the complicated processes of radiotracer transport in blood vessels and extraction into tissues. In our simulation work, we did not employ a true model of blood flow, using only a simulated curve for the generation of BTAC and pulsatile motion of the blood flow, and employing these functions in the simulation. BTACs have been known to have large intrasubject differences for the same radiotracer [39] and therefore rely on many subject factors such as blood pressure, metabolism, and receptor availability. A true mesh model of blood flow of the arteries, veins, and smaller vasculature based on patient-specific imaging such as that described in recent work could be investigated as a method to optimize the arterial velocity profiles [40]. Other rates of arterial velocity of blood in the radial artery (we used 15 cm/s in the wristPET1 simulations) could also be investigated to determine system response for subjects in different physiological states.

The simulated BTAC (Eq. 1) was made from measurements from subjects using an online arterial sampling system as part of a research study with the detector located 1 m away from the extraction site, and not a “true” BTAC (i.e., non-dispersion corrected and measured exactly at the wrist). We have assumed in this work that the measured curve and thus the simulated equation used in the model are marginally affected by dispersion, and although these corrections could be implemented [41], these would not directly impact the outcome of this work. We opted for a simplistic linear-exponential fit to the clinical data, which fit well and proved easy to implement in GATE. There are however more clinically relevant fitting models that could have been investigated.

We also estimated the venous output function, which is less likely to have a sharp peak than the arterial input function, as the bolus of radiotracer undergoes dispersion by passing through the vasculature of the hand and is thus broadened and reduced in peak height. It should also be noted that the arterial plasma concentrations remain independent of the blood sampling site, yet venous concentrations are dependent on the clearance of radiotracer in the vascular bed and thus the sampling site. We have shown that there are mixed arterial and venous signals in the detection process by simulations of 4 deep vessels of the architecture. There are however many more superficial vessels such as the medial anti-brachial vein and cephalic vein, which were not considered in this study. Furthermore, in experimental setups, the device will be unable to distinguish between venous and arterial blood. Figure 6 details contributions to the singles rate from simulations of the venous only, arterial only, and both together. Although we expect a much higher signal contribution from the arterial component during the initial phases of injection, calibration to a gold standard by way of carefully controlled phantoms would be a useful endeavor to account for any bias.

Although this work has not explored imaging capabilities of the system and utilized only count rates, imaging simulations can be performed with the monolithic crystal in order to compare the derived count rates to the image-derived input function (IDIF) of the artery from the dynamic image series. Advantages of the monolithic system include reduced manufacturing costs of designs compared to pixelated crystals with decreasing cross section, a maximized detection efficiency due to reduced dead space, and also direct application of an inherent correction for depth of interaction, the effects of which can be important for PET systems with a small ring diameter such as preclinical or organ-specific systems. Current commercial and research systems have employed monolithic crystals for imaging purposes up to sizes of 50 mm × 50 mm × 20 mm [42, 43]. Potential issues with monolithic crystal imaging include lower signal-to-noise (SNR) due to a wider spread of scintillation light compared to one-to-one coupling. Imaging investigations will also explore the use of trapezoidal crystals, which have been shown to maximize the space within a detector ring [44].

## Conclusions

This work examined the feasibility of a non-invasive in vivo arterial sampling system through the use of computational simulations, showing that on injection of a radiotracer under simulated clinical conditions, the BTAC may successfully be recorded at the wrist using a single half-ring of 8 BGO detectors in coincidence. The sensitivity of the system can be increased by a factor of 10 by the addition of 3 extra detection rings.

## Data Availability

Please contact the corresponding author for the data used in this manuscript.

## Abbreviations

PET: Positron emission tomography
BTAC: Blood time-activity curve
PTAC: Plasma time-activity curve
SNR: Signal-to-noise ratio
IDIF: Image-derived input function
NECR: Noise-equivalent count rate
AIF: Arterial input function
